# New School Meal Regulations Increase Fruit Consumption and Do Not Increase Total Plate Waste

**DOI:** 10.1089/chi.2015.0019

**Published:** 2015-06-01

**Authors:** Marlene B. Schwartz, Kathryn E. Henderson, Margaret Read, Nicole Danna, Jeannette R. Ickovics

**Affiliations:** ^1^ Rudd Center for Food Policy & Obesity, University of Connecticut, Hartford, CT.; ^2^ Henderson Consulting, Guilford, CT.; ^3^ School of Public Policy, University of California Berkeley, Berkeley, CA.; ^4^ Community Alliance for Research and Engagement, Yale University, New Haven, CT.

## Abstract

***Background:*** The 2010 Healthy, Hunger-Free Kids Act required the USDA to update the nutrition standards of the National School Lunch Program. New policies were implemented in the 2012–2013 school year. These changes were followed by anecdotal reports of increased food waste. Empirical research is needed to reliably measure student intake and plate waste before and after this policy change.

***Methods:*** Food consumption and waste was collected annually from a cohort of middle school students in 12 schools in an urban, low-income school district before (spring 2012) and after (spring 2013 and 2014) policy changes. Generalized linear regression was used to compare pre- versus postpolicy selection and consumption of entrées, fruits, vegetables, and milk.

***Results:*** Comparing 2012 to 2014, the percentage of students choosing fruit significantly increased from 54% to 66% and fruit consumption remained high at 74%. Student selection of fruit increased by 9% for each additional type of fruit offered with the meal. The proportion of students who chose a vegetable dropped from 68% to 52%, but students selecting vegetables ate nearly 20% more of them, effectively lowering vegetable waste. Entrée consumption increased significantly from 71% to 84%, thereby also decreasing waste.

***Conclusions:*** Students responded positively to the new lunches. They consumed more fruit, threw away less of the entrees and vegetables, and consumed the same amount of milk. Overall, the revised meal standards and policies appear to have significantly lowered plate waste in school cafeterias.

## Introduction

The National School Lunch Program (NSLP) provides subsidized meals to more than 30 million children every day.^1^ Established in 1946, the NSLP has always required all lunches to meet minimum research-based nutritional requirements.^2^ In recent years, studies of the diets of American children and adolescents have consistently demonstrated the need for an increase in consumption of fruit, vegetables, and whole grains and a decrease in sodium and empty calories from solid fats and added sugars.^3,4^ In response, the federal government took action to update the nutrition requirements of school meals. The Healthy, Hunger-Free Kids Act of 2010 required the USDA to issue regulations to align school meal standards with the 2010 *Dietary Guidelines for Americans*.

The USDA released the proposed rule in January 2011.^5^ Recommended changes included an increase in whole grains, new calorie limits by age group, and a reduction in sodium. Another change was to consider fruits and vegetables two different food categories, require different types of vegetables to be served each week, and increase produce serving sizes. These changes are consistent with research documenting that people consume more when presented with variety and larger portions.^6–8^ The 1981 policy called “Offer vs. Serve”^9^ was updated to address the problem that students do not consume recommended levels of fruits and vegetables. Instead of requiring students to take any three of the five meal components available, the policy was updated to require that one of the three components is a fruit or vegetable serving, thus making the inclusion of a fruit or vegetable with each lunch normative.^10^

The proposed new rules received approximately 130,000 comment letters and the comments were generally supportive; however, one frequently cited concern was the potential increase in plate waste.^11,12^ Specifically, commenters noted that larger portion sizes for fruits and vegetables and requiring students to take a fruit or vegetable would not necessarily lead to increased consumption. Commenters suggested that students may not want the additional food; they do not have enough time to eat a larger quantity of food; and younger students may be overwhelmed by the amount of food. Further, some argued that changing the regulations may lead to lower participation in the program, given that students (particularly older students) may rebel against mandates.^12^

The final rule was released in 2012, and the first phase of changes was implemented in the 2012–2013 school year.^11^ Subsequent to initial implementation of the new regulations, there were anecdotal media reports of an increase in food waste.^13^ Paradoxically, there were also media reports of students saying that there was not enough food served in the new lunches.^14^ To date, there are few empirical studies on student consumption of the new lunches. One study measured plate waste after the new standards went into effect and found that 45% of the food was being thrown away; however, they did not have any prepolicy baseline measures for comparison.^15^ Cohen and colleagues reported prepolicy plate waste rates of 38–43% among middle school students.^16^ In a follow-up study, these researchers compared plate waste data pre- and postregulation change among 1030 school children in four schools in an urban, low-income school district.^17^ They documented postpolicy improvements in both the nutritional quality of the lunch consumed and decreased waste of fruits and vegetables.

Methodologically rigorous studies are needed to evaluate the impact of the Healthy, Hunger-Free Kids Act on food waste in schools over the first 2 years of policy implementation. The aim of this study is to examine food component selection and consumption data from students participating in the NSLP in a low-income, urban district from spring 2012 (preregulation) to spring 2013 and 2014 (postregulation) and measure changes over time.

## Methods

### Participants

Data were collected in 2012, 2013, and 2014 as part of a larger study of student health and academic achievement in an urban school district.^18^ In this district, over 70% of children qualify for free lunch and 13% qualify for reduced-price lunch. The student population is 47% African American, 38% Hispanic, and 15% white. Several years before this study, this school district removed all vending machines and competitive foods from their schools.

Twelve K to eighth-grade schools were randomly selected from the 27 in the district and all agreed to participate. The larger study followed an entire one-grade cohort of approximately 680 students from fifth to seventh grade across the 12 schools. Student BMI was assessed in fifth grade and there was a high prevalence of overweight (19.3%) and obesity (29.9%). The percentage of the cohort who took a lunch during data collection was 80% (*n*=545) in 2012, 75% (*n*=508) in 2013, and 63% (*n*=430) in 2014. The 10% of students who selected an alternative lunch were excluded from analyses owing to our inability to obtain reliable preweights for all of the alternative choices. The final sample included all students who selected the featured school lunch in 2012 (*n*=502), 2013 (*n*=465), and 2014 (*n*=373).

The week before data collection each year, passive consent letters were sent home to all parents describing the protocol and providing the researchers' contact information. The letters explained, “During lunch, we will take a picture of your child's meal tray. This picture will not include your child, only the food and drink items on the tray.” No parents contacted the researchers with questions or to deny consent to this observation study. The school district and the Yale University Institutional Review Board (New Haven, CT) approved all procedures.

### Measures and Procedure

There were a total of 36 data collection days (*i*.*e*., once a year for 3 years for 12 schools). To control for seasonal effects, data were collected each year in April, May, or June.

Before the start of the lunch period, three servings of all available food and beverage items were weighed on a food scale and the average was calculated to serve as the preweight value. After the students swiped their cards with the lunchroom staff, researchers verbally asked the students for permission to take a picture of their trays. None of the children refused. The procedure took only a few seconds and did not disrupt the flow of the line. Trays were numbered sequentially, student gender was recorded, and the trays were photographed. At the conclusion of the meal, research staff collected all lunch trays and weighed and recorded each remaining meal component. Tray photographs were referenced to identify any items that were consumed entirely and left no waste.

Meal components were classified as follows: entrée, fruit, vegetable, and milk. The entrée contained both the grain and meat/meat alternate components. There were 17 different entrées served during the study. No entrée appeared in more than three schools each year of data collection or more than twice in the same school across the years. Juice was separated from the fruit category, so that fruit represented whole fruit or fruit cups. The vegetable component consisted of all vegetables, including potatoes and corn. The milk component included only plain 1% or 2% milk. Flavored milks were not offered during meal times in the district during the 3 years of the study.

### Statistical Analysis

Differences in meal component selection and consumption associated with the change in school meal standards were analyzed using one period of preimplementation data (2012) and two periods of postimplementation data (2013 and 2104). A generalized linear regression model (GLM) was used to analyze differences in both selection and consumption of each meal component: entrée, fruit, vegetable, and milk. Meal component selection was coded as a binary outcome, equal to 1 if one or more servings of the meal component were selected. Meal component consumption was coded as a ratio between 0 and 1, indicating the proportion of the meal component consumed.

To analyze differences in both selection and consumption of each meal component, a GLM was used with a binomial family specification and a logit link function. This method was used to overcome non-normal error distribution and nonlinear effects resulting from the dependent variables being binary or a ratio bounded within the [0, 1] interval. The models control for gender, and cluster robust standard errors were calculated to account for nonindependent observations as a result of repeated measures within schools. A multilevel modeling approach was not used owing to the limited number of schools in the analysis. Average marginal predictions, presented in Tables 1 and 2, were obtained by predicting the average outcome (selection or consumption) for school meal *i* at time *t* and averaging the predictions over all observations for which the model was fitted.

**Table 1.** Meal Component Selection Before and After Implementation of the Updated USDA Standards for School Meals: Marginal Predictions

	Percentage of students selecting item		
	Before implementation	After implementation	
	2012	2013	2014
Meal component	*N*=502	*N*=465	*N*=373
Fruit	53.7 [45.1, 62.2]	70.6^*^ [63.3, 78.0]	66.0^*^ [54.8, 77.2]
Vegetable	68.4 [5S9.4, 77.4]	61.6^*^ [52.4, 70.7]	51.9 [23.4, 80.4]
Entrée	91.4 [86.7, 96.2]	95.5 [91.5, 99.5]	98.3^*^ [96.3, 100.0]
Milk	53.7 [46.8, 60.6]	56.6 [51.0, 62.1]	53.0 [42.2, 63.9]

Asterisks (^*^) indicate significant differences with 2012 at the 5% level. Means calculated using a generalized linear regression model; cluster robust standard errors calculated to account for nonindependent observations. Data in brackets indicate confidence intervals.

**Table 2.** Meal Component Consumption Before and After Implementation of the Updated USDA Standards for School Meals: Estimated Marginal Mean Percentages

	Mean percentage consumed		
	Before implementation	After implementation	
Meal component	2012	2013	2014
Fruit	72.3 [60.5, 84.1] (*n*=269)	60.7 [50.9, 70.6] (*n*=327)	74.3 [69.4, 79.2] (*n*=246)
Vegetable	45.6 [40.5, 50.7] (*n*=344)	38.9 [28.8, 50.0] (*n*=286)	63.6^*^ [53.6, 73.5] (*n*=193)
Entrée	70.9 [59.6, 82.2] (*n*=459)	67.9 [59.3, 76.5] (*n*=443)	83.6^*^ [77.4, 89.8] (*n*=367)
Milk	53.8 [48.5, 59.1] (*n*=268)	53.6 [46.5, 60.8] (*n*=263)	56.7 [48.2, 65.2] (*n*=200)

Asterisks (^*^) indicate significance differences with 2012 at the 5% level. Means calculated using a generalized linear regression model; cluster robust standard errors calculated to account for nonindependent observations. Data in brackets indicate confidence intervals.

## Results

### Selection

Table 1 shows the percentage of students who selected each meal component by year, before and after the implementation of the new school meal standards. The percentage of students selecting a fruit significantly increased after the new standards took effect, from 54% in 2012 to 71% in 2013 and 66% in 2014 (*p*<0.05, for comparisons of both postimplementation periods to baseline). The percentage of students selecting vegetables significantly decreased from 68% in 2012 to 62% in 2013 (*p*<0.05); however, the difference between the 2012 and 2014 means is not statistically significant owing to the degree of variation in the 2014 data. Over half of the students selected milk with their lunches, and this level remained consistent over all 3 years. Whereas nearly all students selected an entrée as one of the three required components all three years, there was a significant rise from 91% in 2012 to 98% in 2014 (*p*<0.05).

### Consumption

Table 2 shows the percentage consumed of each meal component among the students who selected the meal component. The percentage of the vegetable serving consumed did not change significantly the first year of the new standards, but did increase significantly from 45% in 2012 to 64% in the second year, 2014 (*p*<0.05). Consumption of the entrée meal component followed a similar pattern: Levels remained consistent from 2012 to 2013, followed by a significant increase from 71% in 2012 to 84% in 2014 (*p*<0.05). Milk consumption remained consistent over all 3 years, with students consuming approximately half their milk. There were no significant differences in the percentage of fruit consumed; consumption levels ranged from 61% to 74% over the 3 years.

### Variety and Preferences

Over the 3 years, there were a variety of fruits offered to the students. Some schools would offer only one type of fruit per meal, whereas others offered multiple options. An ordinary least squares regression was used to test whether the number of fruit options presented each day influenced the percentage of children who selected fruit at that meal. Holding school and year constant, this test revealed a significant positive relationship between the number of choices and frequency of selection; specifically, increasing the number of fruit options by one is associated with a 9.3% increase in fruit servings selected by students. Further, students exhibited preferences for some produce over others. Table 3 lists the average percent consumed for the most popular fruit and vegetable types, combining the data from all schools and all years.

**Table 3.** Percentage of Meal Component Consumed by Most Popular Fruit and Vegetable Type, Across All Schools and Years

Fruit type	% consumed	Vegetable type	% consumed
Fruit cup	88	Potatoes^a^	72
Banana	78	Corn	65
Orange	70	Beans	46
Pear	56	Salad	42
Apple	48	Broccoli	38

^a^ Potatoes served were not fried.

## Discussion

Our results indicate that the revised NSLP nutrition standards and policies have led to more nutritious meals and less overall plate waste. The increase in fruit selection combined with consistent rates of fruit consumption means that more students are consuming fruit and the percentage of fruit students throw away has not increased as a result of the policy change. There has also been a decrease in vegetable plate waste. Although fewer students are selecting vegetables, those who do choose vegetables eat more of the serving and throw away less. Despite concerns that students do not like the new entrées that meet the whole grain and meat/meat alternate regulations, our data show that more students are selecting the entrée and they are wasting significantly less because consumption is up to 84%.

The increase in fruit selection may, in part, be attributed to an increase in the number of fruit options offered to students postimplementation of the new standards. We found that students enjoy variety and are more likely to choose fruit with each additional option. Interestingly, the fruit cup (which includes different types of fruit, such as pineapple, peaches, and grapes in water, 100% juice, or light syrup) was among the most consumed items.

The findings from our study are consistent with those from Cohen and colleagues.^17^ It is notable that both studies examined children in a low-income, urban district. It is possible that the new school lunches have been accepted more readily in districts where the majority of the students are eligible for free/reduced lunch because the lunch program is viewed as an integral part of the school. It is also possible that low-income students are used to eating the school lunch each day and feel comfortable with the food service in their schools, making them more willing to trust them and try new options. Turner and Chaloupka^19^ recently did a national survey of administrators and food service staff in elementary schools after the USDA regulations went into effect and most reported that students were eating the new lunches, especially those from urban and low-income districts.

A distinctive characteristic of the district in the current study is that it does not offer competitive foods in the cafeteria. It is possible that other districts have seen students switch from the school lunch to competitive foods since 2012. Importantly, the new “Smart Snacks” standards for competitive foods will ensure that all à la carte snack and vending options also meet nutrition standards. This will improve the overall nutrition environment of schools and reduce the problem of school meals having to compete with unhealthy snacks within the building.

The present study has some limitations. Whereas data collection took place over 3 years in the same schools with the same group of children, the design would have been stronger if we had been able to match individual children from one year to the next. This cohort design also introduces the possibility that participants changed their eating behavior as they aged from fifth to seventh grade. It is conceivable that, as students grew older, they also ate more and wasted less. However, if older children consumed more overall, one would expect an increase in milk consumption because it was the meal component that did not change. This did not occur; the selection and consumption of milk was remarkably steady over time. Another possibility is that social desirability influenced student eating because they knew they were being observed. If this were the case, one would expect the effect to be consistent over the years and therefore not influence the primary research questions.

Another limitation of this study is that we collected data only once a year from each school. This creates the possibility that an extremely popular entrée such as pizza could disproportionately influence our findings. Fortunately, this concern is reduced because there were 17 different entrées served across the 36 days and no single option was systematically present in a particular school or year.

An additional limitation is that we do not know why our sample size decreased each year. Unfortunately, we were not able to collect data on the students from our cohort who did not choose the school lunch. We do not know whether they were absent that day, eating a lunch from home or outside of school, or not eating at all. One reason for the decrease may be that, as the students grew older, they are less likely to participate in the school lunch. Other research from Connecticut comparing elementary, middle, and high school lunch participation rates found that participation decreases from one school level to the next.^20^

Another explanation may be that fewer students chose the school lunch each year because they did not like the new options. To explore this possibility, we examined state-wide data and found that participation in the NSLP decreased overall from 2012 to 2014; however, this was preceded by consistent annual decreases from 2010 and 2011 as well, suggesting that the recent decrease cannot be attributed to the new regulations alone.^21^

## Conclusions

This study adds evidence to the scientific literature on student selection and consumption of different components of the school lunch. We had the opportunity to examine selection and consumption before and after USDA regulation updates were implemented. Encouragingly, there was nearly universal acceptance of the new entrée selections, and entrée plate waste dropped significantly after the new standards were implemented. Milk consumption remained the same. The new requirement for students to select a fruit or vegetable with each lunch is an effective strategy to improve the nutritional quality of school meals. There was no evidence of an increase in the percentage of fruit thrown away, and vegetable waste significantly decreased. Overall, this study suggests that the new standards have led to a decrease in school lunch plate waste.

## References

[1] USDA. Child nutrition tables. 2014 Available at www.fns.usda.gov/pd/child-nutrition-tables Last accessed 125, 2014

[2] Gunderson G. History of the School Lunch Program. 2014 Available at www.fns.usda.gov/nslp/history_5 Last accessed 125, 2014

[3] Guenther PM, Dodd KW, Reedy J, et al. Most Americans eat much less than recommended amounts of fruits and vegetables. J Am Diet Assoc 2006;106:1371–1379 16963342 10.1016/j.jada.2006.06.002

[4] Reedy J, Krebs-Smith SM Dietary sources of energy, solid fats, and added sugars among children and adolescents in the United States. J Am Diet Assoc 2010;110:1477–1484 20869486 10.1016/j.jada.2010.07.010 PMC3428130

[5] USDA Food and Nutrition Service. Nutrition standards in the National School Lunch and School Breakfast Program. Vol. 76, No. 9. Vol. 7 CFR Parts 210 and 220. Federal Register 2011:2494–2570

[6] Wansink B, Kim J Bad popcorn in big buckets: Portion size can influence intake as much as taste. J Nutr Educ Behav 2005;37:242–245 16053812 10.1016/s1499-4046(06)60278-9

[7] Zampollo F, Kniffin KM, Wansink B, et al. Food plating preferences of children: The importance of presentation on desire for diversity. Acta Paediatr 101:61–66 21767305 10.1111/j.1651-2227.2011.02409.x

[8] Kahn BE, Wansink B The influence of assortment structure on perceived variety and consumption quantities. J Cons Res 2004;30:519–533

[9] Ralston K, Newman C, Clauson A, et al. The National School Lunch Program: Backgrounds, trends, and issues. Economic research report no. 61. USDA: Washington, DC, 2008

[10] Wansink B. Convenient, attractive, and normative: The CAN approach to making children slim by design. Child Obes 2013;9:277–278 23865529 10.1089/chi.2013.9405

[11] USDA (ed). Nutrition standards in the National School Lunch and School Breakfast Programs: Final rule. FNS 2007-0038. Vol. 7 CFR Parts 210 and 220. Federal Register 2012:4088–4167 22359796

[12] ICF Incorporated. FNS coding and comment analysis: Proposed rule on all food sold in school as required by the Healthy Hunger-Free Kids Act of 2010. Final Summary of Public Comments. 6 21, 2013 Available at www.fns.usda.gov/allfoods_commentsummary.pdf Last accessed 32, 2015

[13] Yee V. No appetite for good-for-you school lunches. The New York Times. 10 5, 2012

[14] Hellmich N. Students push back on new school lunches. USA Today. 9 18, 2012

[15] Byker CJ, Farris AR, Marcenelle M, et al. Food waste in a school nutrition program after implementation of new lunch program guidelines. J Nutr Educ Behav 2014;46:406–411 24857599 10.1016/j.jneb.2014.03.009

[16] Cohen JF, Richardson S, Austin SB, et al. School lunch waste among middle school students: Nutrients consumed and costs. Am J Prev Med 2013;44:114–121 23332326 10.1016/j.amepre.2012.09.060 PMC3788640

[17] Cohen JF, Richardson S, Parker E, et al. Impact of the new U.S. Department of Agriculture school meal standards on food selection, consumption, and waste. Am J Prev Med 2014;46:388–394 24650841 10.1016/j.amepre.2013.11.013 PMC3994463

[18] Ickovics JR, Carroll-Scott A, Peters SM, et al. Health and academic achievement: Cumulative effects of health assets on standardized test scores among urban youth in the United States. J Sch Health 2014;84:40–48 24320151 10.1111/josh.12117 PMC4058503

[19] Turner L, Chaloupka FJ Perceived reactions of elementary school students to changes in school lunches after implementation of the United States Department of Agriculture's new meals standards: Minimal backlash, but rural and socioeconomic disparities exist. Child Obes 2014;10:349–356 25045934 10.1089/chi.2014.0038 PMC4121045

[20] Long MW, Henderson KE, Schwartz MB Evaluating the impact of a Connecticut program to reduce availability of unhealthy competitive food in schools. J Sch Health 2010;80:478–486 20840657 10.1111/j.1746-1561.2010.00531.x

[21] USDA. National School Lunch Program total participation by state. 2015 Available at www.fns.usda.gov/pd/child-nutrition-tables Last accessed 120, 2015

